# The mediating effect of ^18^F-FDG metabolism in right caudate between depressive symptoms and cognitive function in Alzheimer’s disease

**DOI:** 10.3389/fnagi.2024.1328143

**Published:** 2024-03-06

**Authors:** Bojun Wang, Chunlei Cui, Yifan Chen, Zhigang Liang

**Affiliations:** ^1^Department of Radiology and Nuclear Medicine, Xuanwu Hospital, Capital Medical University, Beijing, China; ^2^Department of Radiology, Beijing Bo'ai Hospital, China Rehabilitation Research Center, Beijing, China

**Keywords:** Alzheimer’s disease, depressive symptoms, positron emission tomography/magnetic resonance imaging (PET/MRI), ^18^F-Fluoro-2-deoxy-D-glucose (^18^F-FDG), mediating effect, standardized uptake value ratio (SUVR)

## Abstract

**Purpose:**

The objective of this study was to investigate the accumulation of ^18^F-fluorodeoxyglucose (^18^F-FDG) in the whole brain between Alzheimer’s disease (AD) with depressive (ADD) symptoms compared with AD without depressive (ADND) symptoms using positron emission tomography/magnetic resonance imaging (PET/MRI). Additionally, this study aimed to explore the associations among the accumulation of ^18^F-FDG in the brain, depressive symptoms, and cognitive function in ADD patients.

**Methods:**

In this study, 25 AD patients and 22 healthy controls were enrolled. The AD patients were stratified into two groups, namely ADD and ADND, based on their scores of the Hamilton Depression Scale (HAMD). Both AD patients and healthy controls underwent an ^18^F-FDG PET/MRI scan. A standardized uptake value ratio (SUVR) was calculated to examine the accumulation of ^18^F-FDG in the brain. A simple mediation model was employed to examine the mediation effect between SUVR, depressive symptoms and cognitive function in ADD patients.

**Results:**

The ADD group exhibited significant cognitive impairment compared to the ADND group (*p* < 0.001) and healthy controls (*p* < 0.001). The ADD patients exhibited the reduced SUVR (0.228 ± 0.126) in the right caudate (the voxel level *p* < 0.005, cluster level *p* < 0.05, after false discovery rate (FDR) correction) compared to ADND patients (0.459 ± 0.064) and healthy controls (0.706 ± 0.122). The SUVR of the right caudate was correlated with the HAMD scores (*r* = −0.792, *p* < 0.001) and mini-mental state examination (MMSE) (*r* = 0.738, *p* < 0.01). The relationship between depressive symptoms and the cognitive function in ADD patients is mediated by the right caudate SUVR (total effects = −0.385, direct effects = −0.02, total indirect effects = −0.405).

**Conclusion:**

The ADD group exhibited the reduced SUVR in the right caudate compared to the ADND group and healthy controls. The relationship between depressive symptoms and the cognitive ability of AD patients was mediated by the right caudate SUVR. The results contribute to a deeper understanding of the neurobiological mechanisms related to AD with depressive symptoms.

## Introduction

Alzheimer’s disease (AD) is the most common neurodegenerative disease in the older people, which is characterized by progressive cognitive impairment and behavioral impairment. AD is often accompanied by neuropsychiatric syndrome (NPS). approximately one-third of patients develop NPS early in the course of the AD (Burns et al., 1990). Depressive symptoms are the most common in NPS of AD, accounting for 37–57% of the prevalence of NPSs (Gedde et al., 2021). Depressive symptoms can trigger anxiety, apathy, and somnipathy and increase cortisol levels in the blood through the hypothalamic–pituitary–adrenal axis (HPA). These factors induced cognitive impairment and affected the prognosis (Lyketsos et al., 1999; Bassiony et al., 2002; Orgeta et al., 2017). Therefore, the accurate detection of depressive symptoms in AD patients and timely administration of antidepressant treatment can effectively improve the cognitive function in AD patients.

However, there are no clear criteria to clearly diagnose the depressive symptoms in AD patients (Olin et al., 2002). Neuropsychological scales are commonly employed in clinical settings to assess depressive symptoms in AD patients, but the scores obtained from these scales are susceptible to the patients’ mental state. Neuroimaging has been proven to be useful for the auxiliary diagnosis of depressive symptoms. The ^18^F-flurodeoxyglucose (^18^F-FDG) positron emission tomography (PET) is one of the advanced equipment with certain advantages. The standard uptake value ratio (SUVR) serves as a semi-quantitative index to evaluate the accumulation of ^18^F-FDG in the brain.

Studies revealed that the depressive symptoms of AD patients were related to the dysfunction of multiple brain regions (Hirono et al., 1998; Holthoff et al., 2005; Lee et al., 2006, 2010; Lebedeva et al., 2014; Guo et al., 2015; Du et al., 2023). However, these studies primarily focused on investigating functional changes in specific brain regions of ADD patients (Terada et al., 2014; Dai et al., 2023; Du et al., 2023). There were few studies explore the changes in whole-brain ^18^F-FDG metabolic patterns in ADD patients. Additionally, studies have consistently demonstrated that there is a significant association between depressive symptoms and impaired cognition, although the underlying mechanisms remain unclear (Botto et al., 2022; Acosta-Baena et al., 2023). It is necessary to explore their underlying relationship further.

Therefore, this exploratory study aimed to investigate the accumulation of the ^18^F-FDG in the whole brain between ADD and ADND groups, using PET/MRI, and to explore the associations between SUVR, depressive symptoms, and cognitive function in ADD patients. This study provided some enlightenment on the neurodegenerative mechanism of AD patients with psychiatric symptoms.

## Methods

### Subjects

**Figure 1:**
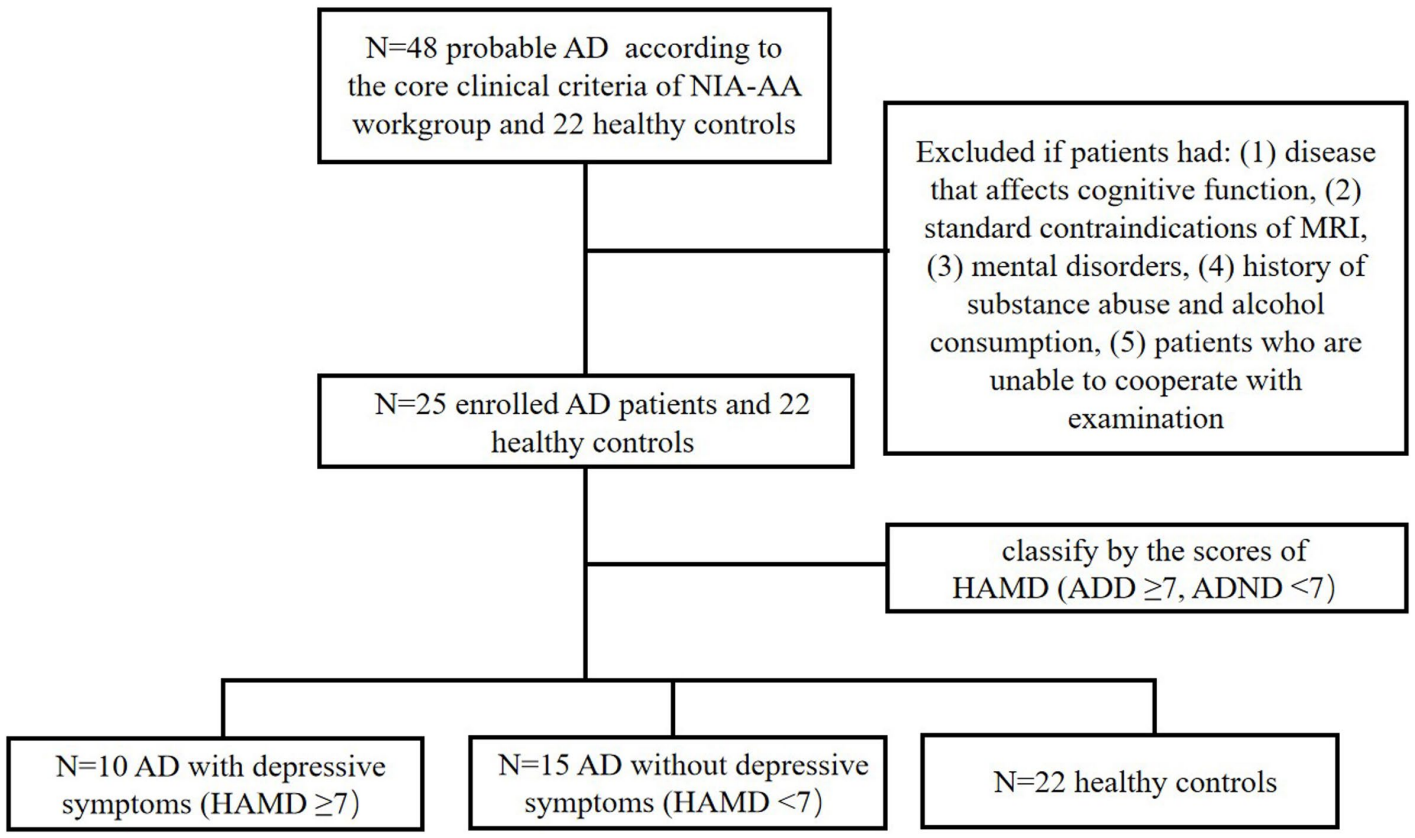
Patient inclusion and exclusion flow diagram.

A total of 47 AD subjects were recruited in this study (Figure 1), including 10 AD patients with depressive symptoms (ADD), 15 AD patients without depressive symptoms (ADND), and 22 healthy controls, all of whom were treatment naive. An effect size calculator was employed to obtain Cohen’s d.^1^ Subsequently, G*Power software was used to estimate the sample size. The statistical analysis demonstrated that a total of 47 subjects were sufficient to provide a power of more than 90% at a 5% significance level.

All AD patients underwent a standard medical evaluation to establish a clinical diagnosis, each evaluation included the following: basic clinical information, a detailed cognitive test battery, and brain ^18^F-FDG PET/MRI scans. All the neuropsychological scales, including the HAMD, were measured by experienced neurologists to eliminate false negatives as much as possible due to the cognitive impairment of the patients. AD was diagnosed according to the core clinical criteria of the National Institute on Aging–Alzheimer’s Association (NIA-AA) workgroup for probable AD (McKhann et al., 2011). Specific eligibility criteria were as follows: 1. an age range of 40–90 years; 2. fulfillment of the core clinical criteria of the National Institute on Aging and the Alzheimer’s Association workgroup for probable AD (the diagnosis of dementia due to Alzheimer’s disease: recommendations from the National Institute on Aging-Alzheimer’s Association workgroups on diagnostic guidelines for Alzheimer’s disease). A. Insidious onset. B. Clear-cut history of worsening of cognition by report or observation. C. Cognitive deficits involvement of a minimum of two of the following cognitive domains: a. amnestic presentation and b. non-amnestic presentations: language, visuospatial, executive dysfunction, etc. D. Decreased ^18^F-FDG uptake on PET in the temporo-parietal cortex. E. Disproportionate atrophy on structural MRI in the medial, basal, and lateral temporal lobe, and the medial parietal cortex. 3. Patients who were diagnosed for the first time without the use of any AD-related medications, including anti-cholinesterase or N-methyl-D-aspartic acid receptor antagonists. 4. Right-handed participants.

Patients were excluded if they had (1) disease that affects cognitive function, such as Lewy body dementia, brain trauma, neurosyphilis, or thyroid dysfunction; (2) concomitant cerebrovascular disease, defined by a history of a stroke temporally related to the onset or worsening of cognitive impairment, or the presence of multiple or extensive infarcts or severe white matter hyperintensity burden; (3) standard contraindications of MRI such as magnetic metal implants or metal dentures; (4) other mental disorders such as anxiety, based on the scores of HAMD; (5) history of substance abuse and alcohol consumption; and (6) patients who are unable to cooperate with examination. HAMD scores were conducted to distinguish between patients with ADD and ADND. The diagnosis of ADD is established when the HAMD score surpasses 7, while a score below this threshold indicates a diagnosis of ADND (Price et al., 2022).

All patients provided informed consent. The study was in line with the newly revised Declaration of Helsinki, and the experimental procedure was approved by the local ethics committee of Xuanwu Hospital in Capital Medical University ([2020]026).

### Neuropsychological assessments

Standard clinical neuropsychological assessments were conducted by a professional neurologist. Mini-mental state examination (MMSE) (Folstein et al., 1975), Montreal Cognitive Assessment (MoCA) (Nasreddine et al., 2005), and Clinical Dementia Rating (CDR) (Morris, 1993) were employed to assess global cognitive abilities. Rey’s auditory-verbal learning test (AVLT) (Bean, 2011) was conducted to evaluate memory function. Digital span test (DST) (Silva et al., 2019) was utilized to assess attentional capacity. Trail making test B-A (TMT B-A) (O’Sullivan et al., 2005) was used to evaluate cognitive functions, principally attention and working memory. Boston naming test (BNT) was conducted to measure confrontational word retrieval abilities in individuals with aphasia and other language disturbances. Activities of daily living (ADL) (Reisberg et al., 2001) assessments were employed to evaluate individual’s ability to perform daily tasks. HAMD (Hamilton, 1960) was used to assess the severity of depressive symptoms.

### Imaging acquisition paraments

Brain ^18^F-FDG PET/MRI scans were conducted using a hybrid time-of-flight (TOF) PET/MRI (Signa PET/MRI, GE Healthcare, WI, United States) system equipped with a 19-channel head and neck union coil. Patients were treated with an ^18^F-FDG radiotracer produced in-house by the Department of Nuclear Medicine. These patients fasted for at least 6 h prior to the examination and had a blood glucose level of <8.4 mmol/L. Patients received an intravenous injection of ^18^F-FDG at a dose of 300–370 MBq/kg and they rested in a quiet, light-protected environment for 40 min until the examination.

The PET/MRI acquisition protocol was in line with the procedure guidelines provided by the European Association of Nuclear Medicine (EANM) (Varrone et al., 2009). Moreover, 3D T1-weighted imaging (TIWI) and ^18^F-FDG PET imaging were acquired simultaneously during the same session. In addition, a FLAIR sequence was also acquired to screen for other brain lesions and abnormalities. Scanning parameters are described as follows: 3D T1WI: repetition time/echo time = 6.9 ms/2.98 ms, flip angle = 12°, inversion time = 450 ms, matrix size = 256 × 256, field of view = 256 × 256 mm^2^, slice thickness = 1 mm, voxel size = 1 × 1 × 1 mm^3^, and 192 slices. ^18^F-FDG PET: PET images were acquired with a 3D list mode. Attenuation correction based on MRI images was completed using an 18-s 2-point Dixon scan (Mainta et al., 2018). Matrix size = 192 × 192, field of view = 350 × 350 mm^2^, pixel size = 1.82 × 1.82 × 2.78 mm^3^, with spatial resolution of 4.5 mm.

### Imaging preprocessing and analysis

The 3D T1WI and PET images were processed using the SPM12 and PETPVE12 toolkits.

The DARTEL method was employed to segment T1WI, isolate the white matter, gray matter, and cerebrospinal fluid, and perform spatial standardization for each tissue, registering it to the Montreal Neurological Institute (MNI) 152 standard space. The PETPVE12 toolkit facilitated the registration of PET images to T1WI images, and the partial volume effect correction was performed using the Muller-Gartner method with the help of segmentation of gray matter, white matter, and cerebrospinal fluid from T1WI images. The intensity of the corrected SUVR images was normalized using cerebellar VOI as a reference. Subsequently, the normalized intensity PET image is registered to the standard space by means of the spatial normalized deformation field of T1WI images. Finally, the SUVR value of the brain area of interest was extracted. Based on the abovementioned preprocessing steps, the PET images, following spatial standardization, underwent smoothing using 8*8*8 mm smoothing nuclei, and the whole brain voxel-by-voxel statistics were conducted on the smoothed image.

### Statistical analysis

Statistics was analyzed by Statistical Package for the Social Science (SPSS) (version 27.0) and SPSS PROCESS (version 4.1 by Andrew F. Hayes)^2^ for Windows.

A group comparison analysis of age, gender, educational years, and neuropsychological assessments was conducted using one-way analysis of variance (ANOVA) followed by Bonferroni’s multiple comparison test and the chi-squared test. Notably, a *p*-value of <0.05 exhibited statistical significance. A two-sample *t*-test was utilized to assess the differences in brain ^18^F-FDG metabolism between ADD and ADND groups. One-way analysis of variance (ANOVA) followed by Bonferroni’s multiple comparison test was employed to analyze the differences in right caudate SUVR among the patients with ADD, and ADND and healthy controls. The significance levels of all cluster analyses were set at a voxel and cluster level of *p* < 0.005 and *p* < 0.05, respectively, following the correction of false discovery rate (FDR). Age, gender, and educational years were used as covariates.

In terms of intermediary effect, initially, a normality test was conducted on continuous numerical variables such as the clinical scale scores and SUVR of the brain regions. Pearson’s and Spearman’s correlation analyses were employed to examine the relationships between SUVR and neuropsychological scale scores across various cognitive domains of ADD patients. Subsequently, an analysis was conducted to ascertain whether SUVR played a significant mediating role in the association between depressive symptoms and the cognitive function among ADD patients. To evaluate the mediation effect, a bootstrapping analysis was conducted with 5,000 repetitions of sampling to calculate a 95% confidence interval (CI) and establish significance. The significance of an indirect mediation effect was determined at the level of 5% by examining whether zero was included in the 95% Bootstrap CI.

## Results

### Participants

In this study, 10 patients with ADD, 15 patients with ADND, and 22 healthy controls were included. Their demographic and cognitive characteristics are shown in Table 1. No significant differences were observed between the three groups in gender, age, and educational years (all *p* > 0.05). The ADD group exhibited inferior performance on mini-mental state examination (MMSE), Montreal cognitive assessment (MoCA), digital span test (DST), Boston naming test (BNT), activity of daily living (ADL), clinical dementia rating (CDR), and the HAMD compared to the ADND group (all *p*<0.05). No significant differences were detected between the groups in the auditory verbal learning test (AVLT) and trail making test (TMT) B-A (*p* > 0.05), although the ADD group exhibited a trend of deterioration compared to the ADND group. Both ADD and ADND groups exhibited inferior performance on MMSE, MoCA, AVLT, DST, TMT B-A, BNT, ADL, CDR, and HAMD (*p* > 0.05).

**Table 1:** Demographic characteristics and neuropsychological scales.

	ADD group	ADND group	Controls	*p*	*p1*	*p2*	*p3*
Age	57.40 ± 6.31	59.47 ± 5.04	55.32 ± 8.29	0.218	–	–	–
Gender (M: F)	5:5	5:10	12:10	0.435	–	–	–
Educational years	11.30 ± 3.74	11.80 ± 3.51	11.27 ± 2.91	0.881	–	–	–
MMSE	10.90 ± 2.96	15.67 ± 4.07	29.36 ± 0.79	<0.001	0.008	<0.001	<0.001
MoCA	6.70 ± 2.50	10.40 ± 3.72	27.27 ± 1.64	<0.001	0.020	<0.001	<0.001
AVLT	6.30 ± 3.23	9.80 ± 3.49	24.91 ± 6.09	<0.001	0.255	<0.001	<0.001
DST	8.70 ± 2.58	10.80 ± 2.01	13.32 ± 1.81	<0.001	0.048	<0.001	0.002
TMT B-A	159.00 ± 22.34	145.60 ± 43.71	37.23 ± 44.9	<0.001	0.693	<0.001	<0.001
BNT	12.30 ± 3.50	18.07 ± 6.61	25.68 ± 22.15	<0.001	0.028	<0.001	0.002
ADL	43.90 ± 9.37	31.00 ± 7.53	20	<0.001	0.006	<0.001	<0.001
CDR	1.90 ± 0.32	1.27 ± 0.46	0	<0.001	0.001	<0.001	<0.001
HAMD	11.70 ± 7.39	3.07 ± 1.94	0.27 ± 0.63	<0.001	0.015	0.003	<0.001

The one-way ANOVA and the Bonferroni post-hoc tests were conducted to compare the materials between each group. The results were considered statistically significant with a *p*-value of < 0.05. *p*1: ADD vs. ADND; *p*2: ADD vs. healthy controls; *p*3: ADND vs. healthy controls. ADD: Alzheimer’s disease with depressive symptoms; ADND: Alzheimer’s disease without depressive symptoms; MMSE: mini-mental state examination; MoCA: Montreal cognitive assessment; CDR: clinical dementia rating; AVLT: Rey’s auditory-verbal learning test; DST: digital span test; TMT B-A: Trail making test B-A; BNT: Boston naming test; ADL: activities of daily living; HAMD: Hamilton Depression Scale.

### ^18^F-FDG pet hypometabolism in the whole brain

**Figure 2:**
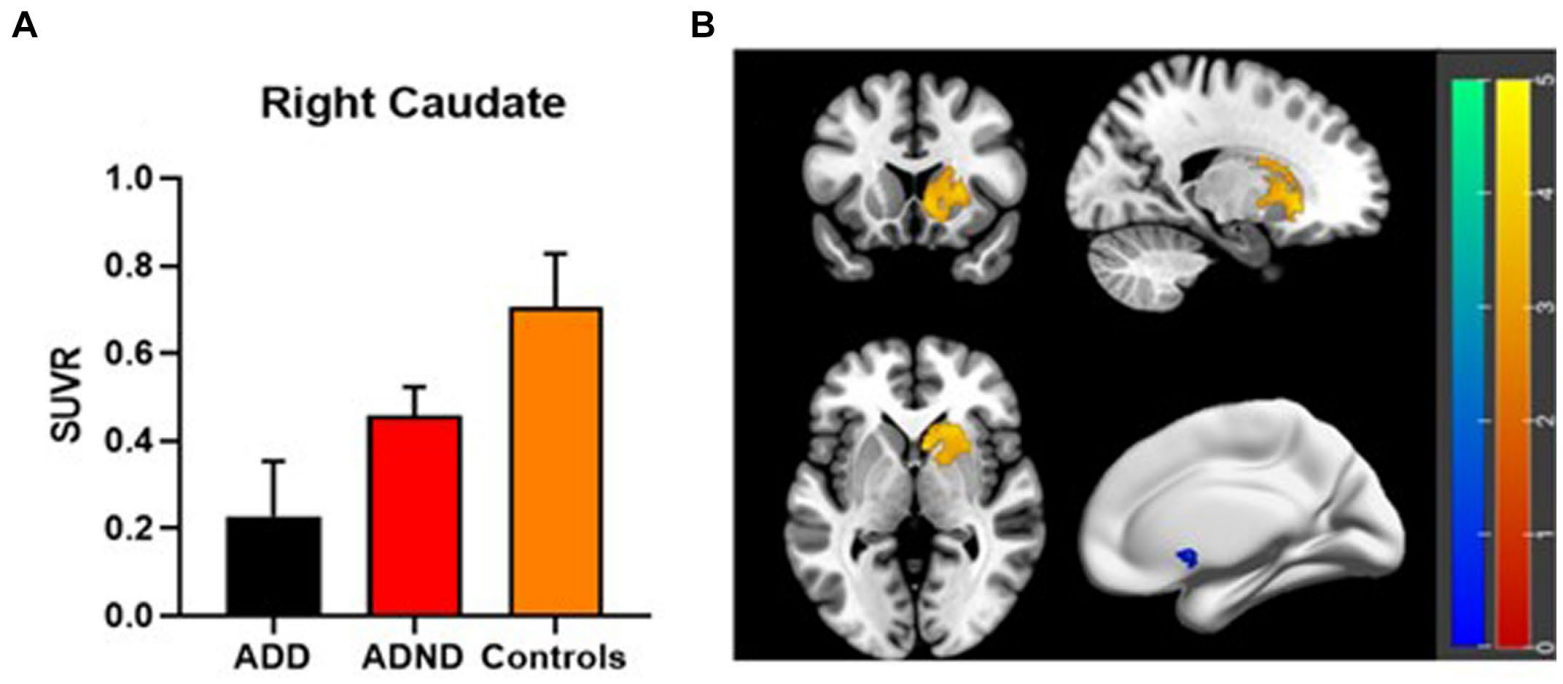
**(A,B)** ^18^F-FDG PET metabolism differences in right caudate among three groups. SUVR: standard uptake value ratio, ADD: Alzheimer’s disease with depressive symptoms, ADND: Alzheimer’s disease without depressive symptoms.

Differences between ADD and ADND groups in the ^18^F-FDG metabolism of the whole brain are shown in Figure 2B; Table 2. A significant decrease occurred in the ^18^F-FDG metabolism of ADD patients was found only in the right caudate region compared to ADND patients (voxel and cluster levels *p* < 0.005 and *p* < 0.05, respectively, following the correction of FDRs). However, no differences were found within other cerebral regions. Additionally, right caudate SUVR in ADD and ADND groups was significantly decreased compared to healthy controls (Figure 2A; Table 3).

**Table 2:** Statistical significance of group difference in ^18^F-FDG metabolism.

Groups	Cluster	MNI			*T* value	Cluster Size
		X	Y	Z		
ADD < ADND	Caudate_R	15	22.5	−7.5	4.7986	2,476

Voxel level with a *p*-value of < 0.005 and cluster level with a *p*-value of < 0.05, after FDR corrected.

**Table 3:** Multiple comparisons after one-way ANOVA.

	ADD	ADND	Controls	*p*	*p1*	*p2*	*p3*
SUVR	0.228 ± 0.126	0.459 ± 0.064	0.706 ± 0.122	<0.001	<0.001	<0.001	<0.001

One-way ANOVA and the Bonferroni post-hoc tests were conducted to compare the materials between each group. The results were considered statistically significant with a *p*-value of < 0.05. p1: ADD vs. ADND; p2: ADD vs. healthy controls; p3: ADND vs. healthy controls. SUVR: standard uptake value ratio.

### Correlation analysis

Bivariate Pearson’s correlation analysis was conducted to test their correlations between depressive symptoms, right caudate SUVR, and general cognitive ability. Correlation coefficients are shown in Table 4. Right caudate SUVR and general cognitive function were negatively associated with depressive symptoms, whereas right caudate SUVR was positively correlated with general cognitive function (r = −0.792, −0.574, and 0.738, respectively). Correlations between the study variables facilitated the construction of a mediation model to elucidate the underlying mechanism of SUVR in depressive symptoms and general cognitive function.

**Table 4:** Correlations of variable.

Variable	1	2	3
Depressive symptoms (HAMD)	–		
Right caudate SUVR	−0.792***	–	
General cognitive function (MMSE)	−0.574**	0.738**	–

Significance was set as *p < 0.05, ** *p* < 0.01, *** *p* < 0.001 (2-tailed). 1: Depressive symptoms (HAMD), 2: right caudate SUVR, and 3: general cognitive function (MMSE).

### The mediating effect of right caudate SUVR

The mediating role of right caudate SUVR between depressive symptoms and cognitive function was tested using a simple mediation mode of the plug-in PROCESS in SPSS. The mediation effect analysis can be employed to construct three regression equations to examine the mediating effect of SUVR on the relationship between HAMD and MMSE (Table 5). The results showed that the direct effect of HAMD on MMSE was not statistically significant following the addition of SUVR variables (β = 0.029, *p* > 0.05). SUVR exhibited a positive effect on MMSE after HAMD was controlled (β = 0.761, *p* < 0.01), whereas HAMD showed a positive effect on SUVR (β = −0.791, *p* < 0.001).

**Table 5:** Testing the mediating effects of SUVR.

	MMSE			SUVR			MMSE		
	SE	*t*	β	SE	*t*	β	SE	*t*	β
HAMD	0.115	−3.359	−0.574	0.003	−6.218	−0.792	0.158	0.125	0.03
SUVR							6.893	3.237	0.762
R^2^	0.329			0.627			0.546		
F	11.283			38.666			13.206		

HAMD: Hamilton Depression Scale; SE, standard error; SUVR: standard uptake value ratio; and MMSE: mini-mental state examination.

**Figure 3:**
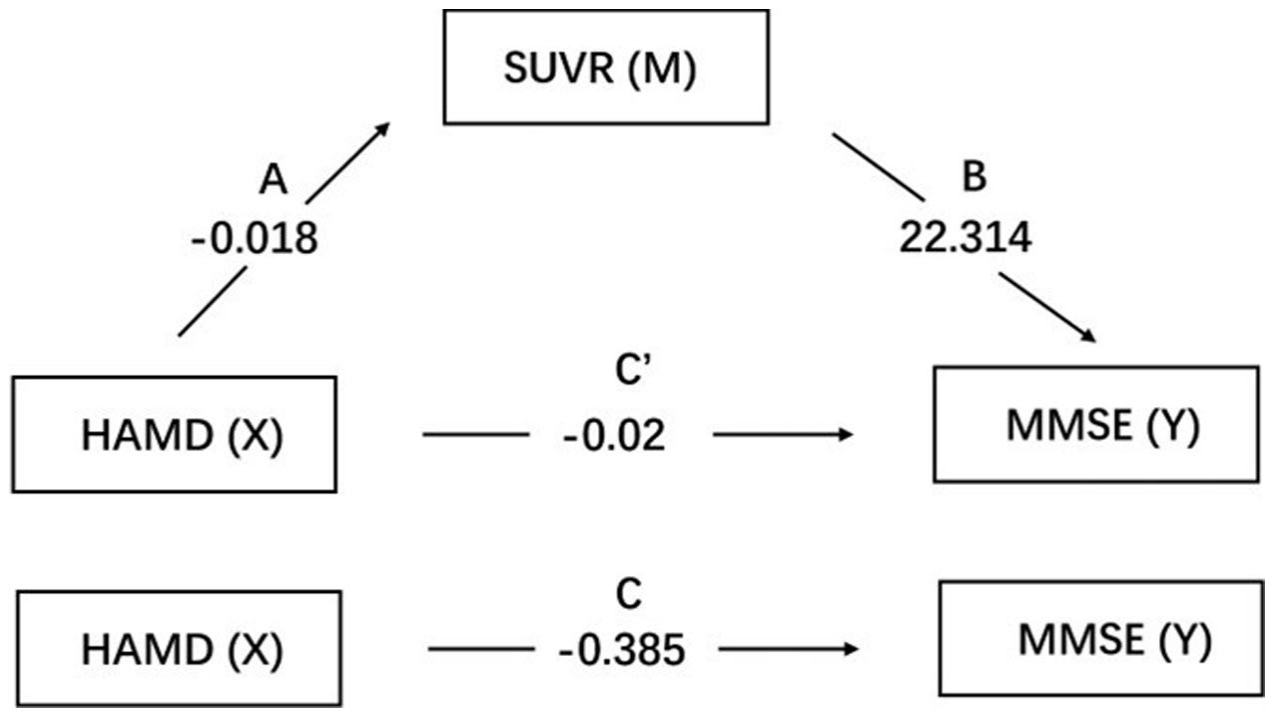
Mediation pathways for SUVR on the association between HAMD and MMSE. Pathways A is the effects of HAMD on SUVR, while B is the effects of SUVR on MMSE. C′ is the direct effect of HAMD on MMSE. SUVR: standard uptake value ratio of right caudate; HAMD: Hamilton Depression Scale; MMSE: mini-mental state examination.

The mediation effect was tested using the Bootstrap method with 5,000 repetitions of sampling. In addition, 95% CI did not include zero for both upper and lower limits, indicating that all the total, direct, and indirect effects of the two mediation paths exhibited statistical significance and were − 0.385, −0.02, and − 0.405, respectively. Coefficients A, B, and C were all significant, whereas C′ was insignificant. The parallel mediation model is shown in Table 6; Figure 3.

**Table 6:** Mediating effects of SUVR on the association between HAMD and MMSE.

Hypothesized mediator	Effect of HAMD on hypothesized mediator		Mediator on MMSE		Mediator on the association between HAMD and MMSE		MMSE		MMSE	
	A (SE)	*P*	B (SE)	*p*	AB (BootSE)	Bootstrap 95%CI	C′ (SE)	*p*	C (SE)	*p*
SUVR	−0.018	<0.001	22.314	0.004	−0.405	(−0.85, −0.173)	−0.02	0.901	−0.385	0.003

CI: confidence interval; SE, standard error; BootSE: standard error obtained based on 5,000 Bootstrap samples; Bootstrap 95% CI: 95% confidence interval generated based on 5,000 Bootstrap samples.

## Discussion

The decreased SUVR was found in the right caudate region in both ADD and ADND groups compared to age-matched healthy controls, while the decreased SUVR was found in the right caudate region only in ADD group compared to the ADND group. Additionally, the mediating effect of the right caudate SUVR on the relationship between depressive symptoms and the cognitive ability of ADD patients was clarified. The association between depressive symptoms and cognitive function in AD was mediated by the SUVR in the right caudate. The findings of this study suggest that depressive symptoms are likely to contribute to cognitive impairment in AD patients by the decreasing SUVR in the right caudate.

The caudate nucleus, as a part of the striatum, plays an important role in the cortico-striato-thalamic-cortical (CSTC) circuit, which serves as an integrated center of brain information processing (Çırak et al., 2020). The caudate nucleus has a central role in cognitive processes and has been implicated in mood disorders (Grahn et al., 2008). Studies shown the dysfunction of right caudate in neurodegenerative disorder-related psychiatric symptom (Douaud et al., 2009; Vriend et al., 2014; Pini et al., 2020), which indicated that the right caudate may be an important node associated with the regulation of psychiatric symptoms. The caudate nucleus is involved in associative functions, motivation, and reward and receives input signals from various regions including prefrontal cortex, hippocampus, and amygdala (Leh et al., 2007; Haber and Calzavara, 2009), suggesting that the dysfunction of the caudate nucleus may affect multiple neural circuits, thereby interacting with diverse psychiatric symptoms observed in patients with neurodegenerative diseases. Functional changes in the caudate can affect the communication pathways between the striatum and other cortices such as the frontal cortex, thereby disrupting the integration of cortical input signals from emotional, cognitive, and motor cortices and producing psychiatric symptoms (Winecoff et al., 2013). Our findings revealed that lower metabolism in the caudate nucleus, as measured by a decline in ^18^F-FDG SUVR, is associated with depressive symptoms and cognition in AD.

In this study, the right caudate SUVR in ADD and ADND groups was significantly decreased compared with age-, gender-, and educational year-matched healthy controls, which suggested that right caudate may exhibit resistance to age-related functional decline. Typical ^18^F-FDG PET findings in healthy controls have manifested reduced glucose metabolism in the frontal and temporal cortex, including lateral orbital gyrus, right temporopola, right orbitofrontal, left orbitofrontal gyrus, left dorsolateral frontal gyrus, and left insula areas (Kim et al., 2009; Subtirelu et al., 2023). These studies indicated that the right caudate may be more susceptible to neuropsychiatric symptoms, which further leads to the reduction in the metabolic function by affecting the circuit function, and may be closely related to the cognitive impairment in ADD patients.

Subsequently, the mediating role of right caudate SUVR between depressive symptoms and cognitive ability was tested, which was discovered that the right caudate SUVR completely mediated the connection between depressive symptoms and the cognitive function in ADD. The caudate is an integral component of the cortico-striatal-pallidal-thalamic loop, which is widely believed to facilitate reward-seeking behavior and the anticipation and assessment of rewarding stimuli (Gold, 2003). Compelling evidence indicates a correlation between caudate dysfunction and the completion stage of the reward process, as well as anhedonia, a cardinal symptom among individuals with depressive symptoms (Pizzagalli et al., 2009), which suggests the essential role of caudate in depressive symptoms. On the other hand, the MRI-based study showed that the caudate is related to an executive function (Macfarlane et al., 2013). This relationship is not unexpected, as the dorsolateral prefrontal cortex generates a prefrontal circuit that facilitates the execution of cognitive function through its interaction with the caudate (Alexander et al., 1986). Of note, a previous neuropsychological study revealed an indirect influence of depressive symptoms on global cognitive impairment, mediated by working and episodic memories, attention and executive function (Yatawara et al., 2016). The aforementioned finding is in line with the proposed role of ^18^F-FDG metabolism of the right caudate in mediating the association between depressive symptoms and cognitive impairment.

### Limitations

Current findings should be interpreted with caution due to a few inherent limitations. First, the small sample size represents a major limitation of this study. Future studies should aim to increase the sample size in order to enhance the statistical power and generalizability of findings. Second, it is essential to acknowledge that the limitations of individual rating scales and the intricate nature of depressive symptoms in AD may restrict the comprehensive assessment of depressive symptoms solely through HAMD. Third, the lack of amyloid and tau level assessment in our study hinders our capacity to determine whether the metabolic changes we observed are directly related to amyloid and tau pathologies. This aspect would enable a more detailed exploration of the relationship between these biomarkers and ^18^F-FDG metabolism, particularly in patients with AD and depressive symptoms. Such studies could provide deeper insights into the disease mechanisms and potentially aid in the development of more targeted therapeutic strategies.

## Conclusion

In conclusion, the right caudate SUVR plays a crucial role in mediating the impact of depressive symptoms on the cognitive impairment of AD patients. These results contribute to advancing the understanding of the intricate association between depressive symptoms and cognitive function, particularly from the perspective of caudate function. Furthermore, these findings lay the groundwork for enhancing cognitive function through targeted interventions based on caudate biomarkers, thereby providing potential avenues for personalized AD treatments.

## Data availability statement

The raw data supporting the conclusions of this article will be made available by the authors, without undue reservation.

## Ethics statement

The studies involving humans were approved by Institutional Review Board of Xuanwu Hospital, Capital Medical University. The studies were conducted in accordance with the local legislation and institutional requirements. The participants provided their written informed consent to participate in this study.

## Author contributions

BW: Conceptualization, Formal analysis, Methodology, Software, Writing – original draft. CC: Conceptualization, Data curation, Methodology, Writing – original draft. YC: Data curation, Investigation, Writing – original draft. ZL: Supervision, Writing – review & editing, Project administration, Resources, Validation.

## Footnotes

^1^ https://www.campbellcollaboration.org/escalc/d-means-sds.php

^2^ http://www.processmacro.org/index.html
